# Bioengineered Pancreatic Cancer Immunosuppressive Microenvironment Models for Screening Immunotherapies

**DOI:** 10.1002/adhm.202502758

**Published:** 2025-11-28

**Authors:** Maria V. Monteiro, Margarida Henriques‐Pereira, Bruno M. Neves, Vítor M. Gaspar, João F. Mano

**Affiliations:** ^1^ Department of Chemistry CICECO – Aveiro Institute of Materials University of Aveiro Campus Universitário de Santiago Aveiro Portugal; ^2^ Cellularis Biomodels Pci Creative Science Park Aveiro Region Via do Conhecimento Edifício Central Ílhavo Portugal; ^3^ Department of Medical Sciences and Institute of Biomedicine – iBiMED University of Aveiro Aveiro Portugal

**Keywords:** immunosuppressive microenvironment, immunotherapy, in vitro models, pancreatic cancer, tumor‐stroma interactions

## Abstract

Pancreatic cancer is notably resistant to treatment, primarily due to its dense desmoplastic stroma and immunosuppressive microenvironment. Accurately modeling this complex landscape and its immunosuppressive hallmarks in vitro is highly valuable for screening immunotherapeutic strategies. However, replicating these intricate features remains a significant challenge. Herein, we bioengineered miniaturized tumor‐stroma platforms that combine cancer and stromal cells, as well as extracellular matrix mimetic biomaterials as a strategy to emulate the native tumor composition and key tumor immunosuppressive signatures. Bioengineered stratified tumor‐stroma pancreatic cancer models, so termed cancer‐on‐a‐bead platforms are generated in superhydrophobic surfaces and co‐cultured with T cells, dendritic cells, as well as M0 macrophages, as a strategy to recapitulate tumor‐immune interplay. The generated models revealed suppression of antigen presentation, M2 macrophage polarization, and T cell exhaustion, representing key features of this neoplasia. The screening of antibody mediated immunotherapy in the 3D tumor platforms, using clinically approved anti PD‐1 antibody as a model therapeutic, partially restored T cell function. Overall, our findings demonstrate compartmentalized tumor‐stroma models potential for being used to screen candidate immunotherapeutics for pancreatic cancer in a preclinical setting.

## Graphical abstract

Bioengineering tumor‐stroma pancreatic cancer models with potential to emulate the native immunosuppressive tumor microenvironment. Cancer‐on‐a‐bead models exhibited higher relevance for screening immunotherapies and modulating the protumoral PDAC microenvironment.

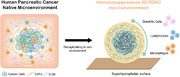

## 1 Introduction

Pancreatic ductal adenocarcinoma (PDAC) remains a highly challenging tumor to treat, as consequence of its late‐stage detection and the limited efficacy of current treatments [1]. PDAC features a highly immunosuppressive and fibrotic microenvironment, dominated by abundant extracellular matrix (ECM) deposition [2, 3]. This ECM is mainly produced by cancer‐associated fibroblast (CAFs), key players in driving tumor resistance and progression [4]. PDAC is also characterized by its unique immune landscape, composed by excessive amounts of protumoral immune cells including myeloid‐derived suppressor cells (MDSCs), tumor‐associated macrophages (TAMs), and regulatory T cells (Tregs) [5, 6]. These cell populations work synergistically to suppress antitumoral activity of cytotoxic T lymphocytes and natural killer cells, thereby promoting an immunosuppressive environment ultimately enabling tumor immune evasion and progression [7]. Additionally, a growing body of evidence highlights the dynamic crosstalk between CAFs and immune cells, as a major contributor to the immunosuppression and resistant nature of this malignancy [5, 8]. CAFs, the main stromal players in pancreatic tumor microenvironment (TME), secrete a number of immunomodulatory agents, including CXCL12, IL‐6, and TGF‐β, that not only promote ECM deposition and remodeling but also immune exclusion [4, 9, 10]. For instance, CXCL12 secretion can contribute to the establishment of a chemokine‐rich barrier that impedes T cell infiltration within the TME [11]. From another perspective, other CAF‐derived factors contribute for the recruitment and polarization of protumoral immune cell populations, such as MDSCs, Tregs, and M2‐type TAMs. Concurrently, evidence demonstrate that CAFs inhibit dendritic cell (DCs) maturation and suppress T cell priming and effector function, reinforcing the immunosuppressive TME [12, 13, 14]. This intricate interplay is central to sustaining tumor fibrosis and promoting immune evasion, ultimately contributing to the failure of current immunotherapeutic strategies.

Envisioning alternatives to current treatment options and aiming to ablate the protumoral immune system, immunotherapeutic approaches have been explored in a number of human solid cancers. However, despite increased efforts to identify immunological targets in PDAC and manipulate its immunosuppressive mechanisms, such as through immune checkpoint inhibitors (e.g., anti PD‐1/PD‐L), and cytokine‐based therapies, clinical outcomes have remained disappointing [5]. These challenges underscore the urgent need for better understanding the intricate PDAC TME landscape and how it affects tumor‐immune interactions, immune exclusion, and suppression. Unraveling these complex dynamics may reveal novel immunomodulatory targets and enable the development of more effective strategies to sensitize PDAC to immunotherapy, ultimately paving the way for improved patient outcomes.

Preclinical tumor models that closely emulate native composition remain of crucial interest for advancing our understanding of the tumor‐immune landscape and identifying potential targets to disturb the immunosuppressive microenvironment [15]. There is still a lack of 3D in vitro models that accurately recreate tumor‐immune interactions [16]. Currently used platforms rely on genetically engineered mouse models or patient‐derived xenografts. To provide more physiologically relevant models, researchers have increasingly focused on bioengineering stratified, compartmentalized platforms capable of more faithfully reproducing the native PDAC tumor composition and bioarchitecture [17, 18, 19]. Leveraging on this, previously established stratified tumor‐stroma, namely cancer‐on‐a‐bead platforms, were characterized for their immunosuppressive properties. Tumor–immune cell interactions were further assessed by co‐culturing these artificial microtumors with various immune cell populations. Specifically, DCs, macrophages, and T lymphocytes were introduced to PDAC microtumors aiming to understand the dynamic tumor‐immune cells interplay. Such an approach aims to better understand how 3D tumor‐stroma models influence immune cells and ultimately identify novel therapeutics targets that could help to ablate or bypass the immunosuppressive TME for more effective treatment strategies for this neoplasia.

## 2 Results

### 2.1 Modeling Pancreatic Tumor Microenvironment in a 3D Setting

**FIGURE 1:**
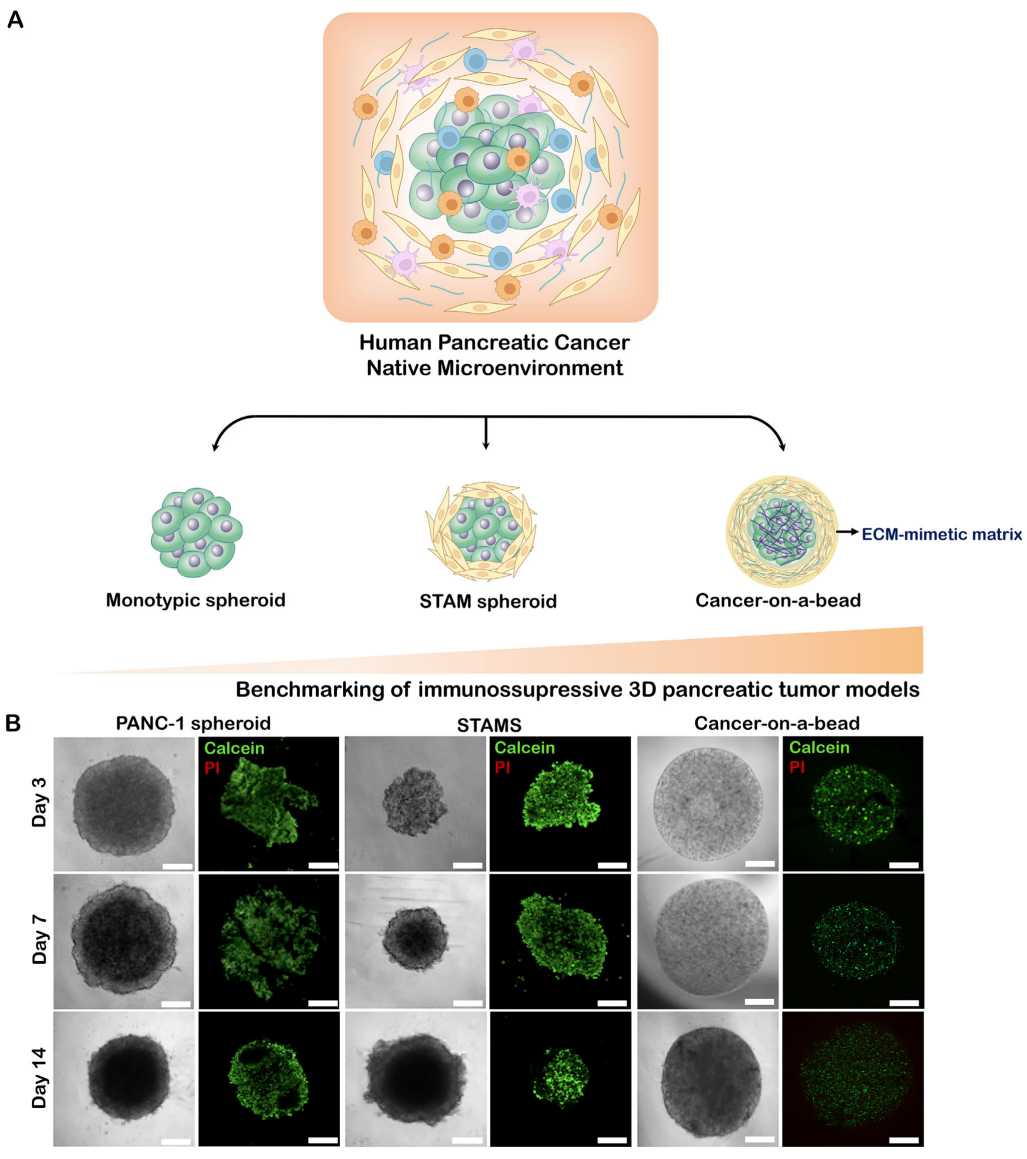
Reproduction of pancreatic cancer TME through 3D in vitro models. (A) Schematics of different miniaturized PDAC 3D platforms, namely PANC‐1 monotypic spheroids; stratified tumor‐stroma STAMS models including cancer cells and CAFs, devoid of external ECM; and cancer‐on‐a‐bead models comprising not only cancer cells and CAFs, but also a ECM‐mimetic hydrogel, where the tumor core bead comprises GelMA 5% (w/v) and the stroma compartment includes GelMA 5% (w/v) and HAMA 1% (w/v). (B) Representative optical micrographs and widefield fluorescence micrographs of live/dead assays of monotypic PANC‐1 spheroids, STAM spheroids, and stratified pancreatic cancer‐on‐a‐bead units at days 3, 7, and 14 of culture. Green channel: Calcein‐AM, red channel: PI. Scale bars = 500 and 200 µm, respectively.

To interrogate the stroma contribution in pancreatic cancer immunosuppression, previously established organotypic pancreatic tumor‐stroma platforms, designed to more closely emulate pancreatic tumor bioarchitecture and composition, were used to study tumor–immune cells interactions and evaluate their potential to screen immunotherapies. For this purpose, cancer‐on‐a‐bead models were fabricated [18]. Such biomimetic tumor‐stroma model is designed to comprise cancer cells cultured in a gelatin methacrylate (GelMA) tumor core bead, surrounded by the stroma compartment, where CAFs are supported by a GelMA‐hyaluronic acid methacrylate (HAMA) hydrogel (Figure 1A). As control models, (i) monoculture PANC‐1 spheroids only comprising cancer cells, and (ii) stratified pancreatic spheroids, also termed as STAMS, where cancer cells are enveloped by CAFs, without the existence of pre‐existing ECM, were established [17]. Such systems allow not only to recapitulate tumor composition, but also, and importantly, its architecture where cancer cells are surrounded by the fibrotic stroma.

Following 14 days of in vitro culture, all 3D PDAC models maintained structural integrity, demonstrating their stability over extended culture periods (Figure 1B). Notably, the heterotypic cancer‐on‐a‐bead constructs exhibited the greatest uniformity in microtumor dimensions, with substantially reduced inter‐unit variability. This high level of consistency underscores the reproducibility of the cancer‐on‐a‐bead system and supports its utility as a robust and scalable platform for high‐throughput therapeutic screening.

Moreover, both heterotypic 3D PDAC models viability outperformed the monotypic model, highlighting the essential role of CAFs in sustaining tumor cell survival (Figure 1B). Yet, cancer‐on‐a‐bead model offers superior physiological relevance by integrating an ECM‐mimetic scaffold, that not only enhances structural fidelity but also imposes a diffusion barrier that limits oxygen, nutrient transport, and active agents, such as therapies, thereby replicating the hypoxic conditions characteristic of the in vivo PDAC microenvironment. This hypoxia‐driven stress contributes to the establishment of an immunosuppressive milieu, ultimately attenuating antitumor immune responses and promoting immune evasion [20].

This dual effect, replicating both the biomechanical architecture and immunological constraints of the native tumor niche, hypothesize the cancer‐on‐a‐bead model as a physiologically and immunologically relevant platform for investigating tumor–immune interactions. These features position it as a powerful tool for dissecting immunosuppressive mechanisms and for the preclinical evaluation of candidate immunotherapies.

### 2.2 Heterotypic Stratified PDAC Models Exhibit Strong Immunosuppressive Gene Signatures

**FIGURE 2:**
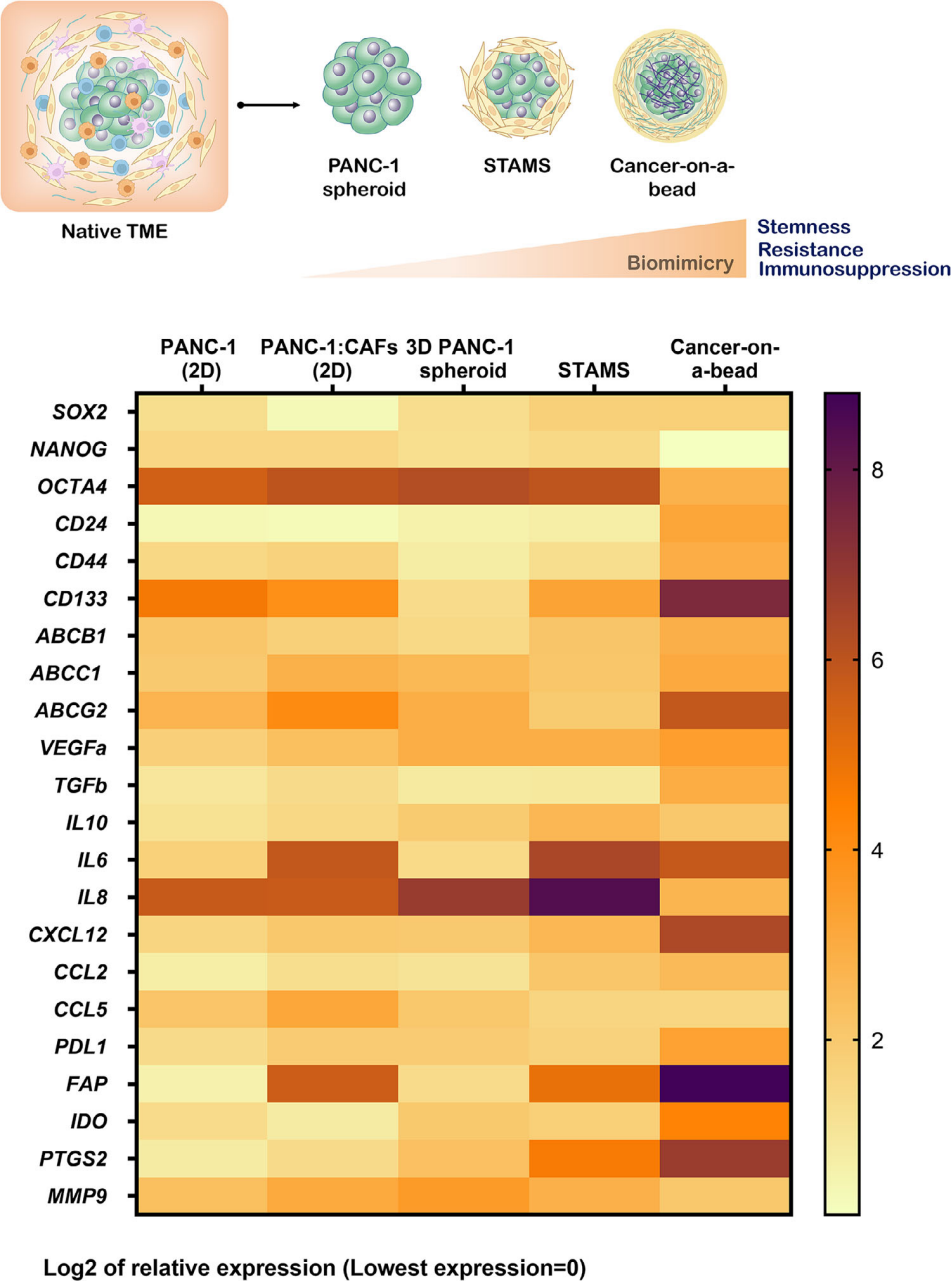
Gene transcriptional signature of tumor‐stroma 3D in vitro models. Total mRNA was extracted from the different models, reverse transcribed, and the transcriptional levels of key immunoregulatory and stemness‐related genes were analyzed by quantitative PCR (qPCR). Log2 mRNA fold changes for each gene were calculated relative to the maximum transcription levels observed across analyzed samples after normalization with reference gene HPRT1. The heatmap displays the mean values from three independent experiments.

CAFs are crucial players of PDAC stroma, contributing significantly to the extensive ECM deposition that surrounds and supports cancer cells [21]. The established dense matrix surrounding tumor mass acts as protective shield ultimately contributing to tumor immune escape and progression [13]. Compartmentalized tumor‐stroma PDAC models incorporating CAFs demonstrated upregulation of desmoplasia‐related genes, matrix metalloproteinases, and immunomodulatory molecules (Figure 2). In fact, the inclusion of CAFs recreates the fibrotic and immunosuppressive microenvironment of PDAC, with elevated transcription of genes coding for key signaling factors such as transforming growth factor—β1 (*TGF‐B1*) and stromal cell‐derived factor 1α (*CXCL12*) (Figure 2). These agents impact angiogenesis, epithelial to mesenchymal transition (EMT), tumor growth and metastasis [3, 22, 23]. Notably, stromal cells addition to the system further increased the transcription of *CXCL12*, interleukin *6 (IL‐6)*, and interleukin 8 (*IL‐8)*, all of which have an immunomodulatory role. These cytokines and chemokines contribute to shaping the TME by recruiting MDSCs, TAMs, and Tregs, thereby facilitating immune evasion and progression.

To further dissect the stromal contribution, CAFs cultured as 2D monolayers, CAF spheroids, and CAFs‐on‐a‐bead were compared. RNA transcripts were analyzed by qRT‐PCR for*IL‐6)*, cyclooxygenase‐2 *(PTGS2), CXCL12, TGF‐B1, CCL2*, fibroblast activation protein *(FAP)*, and programmed cell death 1 ligand 1(*PDL1)* expression (Figure S1). A marked upregulation of IL‐6 and *PTGS2* in CAFs‐on‐a‐bead model was observed, suggesting that CAFs encapsulation in a biomimetic ECM matrix enhances their inflammatory and immunosuppressive phenotype. *CXC12* transcription also increased, consistent with the enhanced *CXCL12* levels observed in cancer‐on‐a‐bead model. *TGF‐B1* and *FAP* transcription remained relatively constant across all conditions, whereas *PD‐L1* levels were higher in 2D CAFs (fold change = 19.26 ± 3.29), whereas expression moderately decreased in spheroids (fold change = 1.34 ± 0.32) and CAF‐on‐a‐bead (fold change = 7.73 ± 1.48), suggesting that the increased *PD‐L1* mRNA levels observed in cancer‐on‐a‐bead platform primarily originates from PANC‐1 cancer cells. Additionally, *CCL2* transcripts were increased in CAFs‐on‐a‐bead, pointing to a key mechanism through which stromal cells may promote immune evasion. This finding aligns with recent studies identifying the CCL2‐CCR2 chemokine axis as a central driver of immunosuppressive macrophage recruitment and T cell exclusion in PDAC [24, 25]. In fact, blockade of CCR2 has been shown to restore antitumor immunity and improve responsiveness to chemotherapy in preclinical PDAC models, with early clinical data confirming the translational potential of targeting this axis [26]. Similarly, human PDAC‐associated cells produce CCL2, promoting infiltration of CCR2^+^ monocytes and correlating with poorer patient survival in tumors displaying high CCL2 expression an low CD8^+^ T vell infiltration [24, 25]. Overall, our results reinforce that cancer‐on‐a‐bead models faithfully reproduce the CCL2‐CCR2‐driven immunosuppressive axis, providing a relevant in vitro testing platform for evaluating future therapeutic interventions targeting this pathway.

From another point of view, the presence of a dense, hyaluronic acid‐rich stroma not only contributes to tissue rigidity, but also acts as a physical and biochemical barrier to immune‐cell infiltration, limiting the diffusion of cytokines, nutrients, and therapeutic agents [27, 28, 29]. Importantly, the characteristic hyaluronan accumulation in PDAC stroma and ECM remodeling are closely linked to CAF activity, as CAFs are major producers of ECM components [9, 30, 31]. This dynamic interplay between CAFs and ECM reinforces a feed‐forward loop of fibrosis and immune exclusion, in which stromal fibrosis hinders cytotoxic T cell penetration and sustains a tumor‐protective niche [32, 33].

Interestingly, tumor‐stroma 3D PDAC models, particularly those incorporating the external supporting ECM —*cancer‐on‐a‐bead*— generally showed a marked upregulation of tumorigenic, stemness‐associated, and immunomodulatory markers. Elevated mRNA levels of *CD24*, *CD44*, *CD133*, *ABCB1*, and *ABCG2* suggests an enrichment of cancer stem‐like cells and a drug‐resistant phenotype, resembling the aggressive and resistant nature of this neoplasia (Figure 2). Concurrently, protumorigenic and immunosuppressive genes, such as *VEGF*, *TGF‐B1*, *IL‐6*, and *PD‐L1* are also upregulated, especially in cancer‐on‐a‐bead models, indicating enhanced angiogenic signaling, EMT and immune evasion.

The presence of stromal activation markers as *FAP*, as well as metabolic and immunomodulatory mediators, such as idoleamine 2,3‐dioxygenase (*IDO*), and *PTGS2*, further supports the bioengineering of a highly immunosuppressive and proinflammatory TME. In particular, TGF‐β, IL‐6, interleukin 10 (IL‐10), and IDO suppress dendritic cells differentiation, maturation, and antigen‐presenting function, driving to a tolerogenic state [34]. Consequently, in the presence of this microenvironment DCs can remain immature and poorly immunogenic, contributing to defective T cell priming [35]. In another hand, these factors promote the recruitment and polarization of macrophages, toward alternatively activated M2 phenotypes, which are reported to enhance immune suppression [36]. CAFs derived molecules such as TGF‐β, FAP, and IL‐6 reinforce this immunosuppressive macrophage profile.

In the context of T cells, exposure to such a TME is expected to impair proliferation, leading to exhaustion and anergy, primarily due to elevated PD‐L1 expression and the presence of immunosuppressive factors, such as TGF‐β and IDO. Overall, the inclusion of stromal components, such as CAFs and exogenous ECM supports the establishment of a tumor TME that promotes the expression of key immunosuppressive molecules. This results in a more physiologically relevant model that closely recapitulates native PDAC features, while also serving as a valuable platform for studying the immunomodulation of immune effector cells, including DCs, macrophages, and T cells.

### 2.3 Impact of PDAC 3D TME on Dendritic Cells Status

Dendritic cells (DCs) are professional antigen‐presenting cells (APCs) functioning as critical orchestrators of the antitumoral immune response [37]. Within the tumor microenvironment, immature dendritic cells (iDCs) capture apoptotic cancer cells, process tumor antigens, and present them on MHC class I and II molecules. During this process, DCs transition from an immature to a mature state, marked by the upregulation of costimulatory molecules and MHC class I and II. In their mature state, DCs migrate to nearby draining lymph nodes, where they present tumor antigens to naïve T cells. This interaction primes tumor antigen‐specific CD4^+^ and CD8^+^ T cells, initiating an adaptive immune response and activating NK cells [37]. However, in a highly immunosuppressive TME, such as that found in human PDAC, DCs become locked in or revert into a tolerogenic state, marked by reduced expression of costimulatory and antigen‐presentation molecules. This scenario compromises their ability to prime effector T cells leading to T cell anergy, expansion of Tregs, and the promotion of tumor immune tolerance. Notably, in PDAC, DCs are subpopulated, being spatially excluded from the juxtatumoral regions, further limiting their interaction with tumor cells and sustaining immune evasion [38].

**FIGURE 3:**
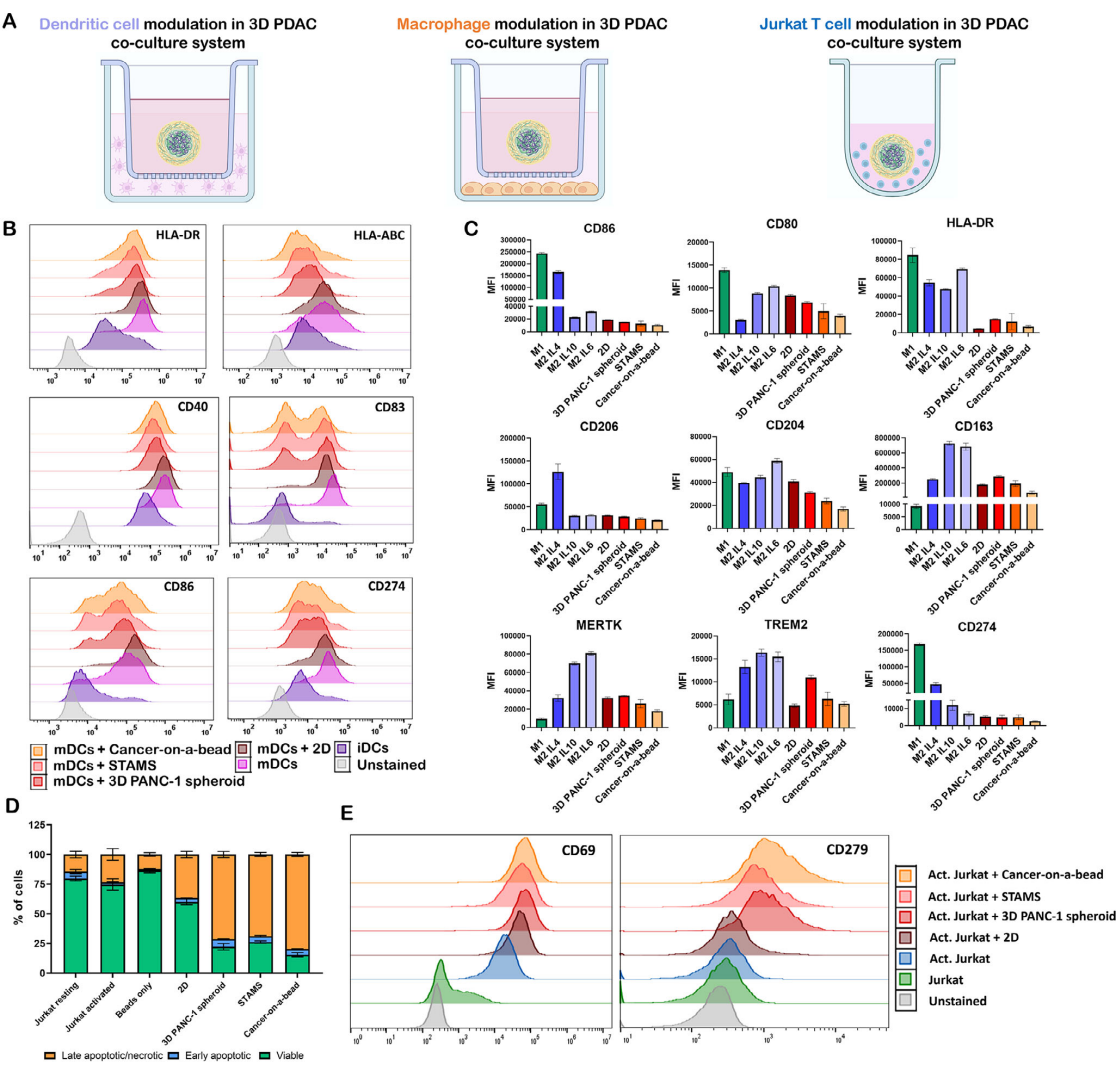
Evaluation of immunomodulatory effects of tumor‐stroma 3D in vitro models on immune cells. (A) Schematics representation of co‐culture 3D PDAC models DCs, macrophages and T lymphocytes. (B) Phenotypic analysis of DCs after co‐culture with 3D in vitro models. Immature DCs were co‐cultured with different tumor models for 24 h and then IL‐1β and TNF‐α were added to the system for additional 24 h to induce DC maturation. DC expression of maturation markers HLA‐DR, HLA‐ABC, costimulatory CD40, CD83, CD86, and co inhibitory CD274 were analyzed by flow cytometry. The displayed histograms represent one experiment, typical of three independent experiments conducted. (C) Analysis of macrophages polarization phenotype. M0 macrophages were co‐cultured with the different tumor models for 48 h and then the expression of key M1 and M2 markers were analyzed by flow cytometry. The phenotype is compared to prototypical M1, M2‐IL‐4, M2‐IL‐10, M2‐IL‐6 ‐polarized cells. Expression levels are presented of mean fluorescence intensity (MFI) of 3 independent experiments. (D–E) Impact of tumours on lymphocyte exhaustion and viability. Activated Jurkat cells, used as surrogates of T lymphocytes, were co‐cultured with different PDAC platforms and apoptotic/necrotic state, and (E) expression of exhaustion markers were addressed by flow cytometry. Results are representative of 3 independent experiments.

To investigate the immunosuppressive effects of 3D PDAC microenvironment on DCs immunostimulatory status, iDCs were co‐cultured with tumor models for 24 h, followed by exposure to maturation agents IL‐1β and TNF‐α for an additional 24 h (Figure 3A). Co‐culture with 3D tumor models resulted in marked downregulation of key maturation and activation markers, including CD86, CD40, CD83, CD247, and HLA‐DR, suggesting a tumor‐induced immunosuppressive effect on DCs (Figure 3B and Figure S2). Particularly, the downregulation of CD80 and CD86 is of great relevance, as it impairs the capacity of DCs to provide the primary costimulatory signal, which is necessary to T cell activation, proliferation, and differentiation into effector T cells [39]. This scenario directly inhibits T cell activation and suppresses adaptive immune responses and antigen presenting ability. These findings align with the observed upregulation of TGF‐β, IDO, and IL‐10 transcription, both well‐established inhibitors of DC maturation, in our tumor‐stroma platforms (Figure 2). The accumulation of TGF‐β, IDO, and IL‐10 affects the regulation of costimulatory agents, such as CD80, CD86, and CD40, as well as MHC class II molecules, which are critical for effective antigen presentation. Additionally, we observed elevated mRNA levels of *PTGS2* in the transcriptional signature of STAMS and cancer‐on‐a‐bead models. The *PTGS2* gene encodes cyclooxygenase‐2 (COX‐2), the key enzyme in prostaglandin biosynthesis, particularly prostaglandin E2 (PGE2). PGE2 upregulates CCR7 expression in DCs, promoting their migration to lymph nodes [40]. However, it significantly inhibits IL‐12 production, a key cytokine essential for polarizing and activating cytotoxic CD8^+^ T cells and supporting Th1 cell function [41]. Recent studies demonstrate that tumor‐derived PGE2 induces a dysfunctional state in intratumoral cDC1s, impairing their ability to locally coordinate antitumor CD8^+^ T cell responses [42].

From a broader perspective, our 3D tumor models have proven to be effective platforms for studying DC‐tumor interactions, particularly in developing DC‐based immunotherapies. These include strategies to enhance DC infiltration and survival within the TME or to engineer DCs resistant to tumor‐derived immunosuppressive signals.

### 2.4 Stratified Tumor‐Stroma PDAC 3D Models Polarize Macrophages Through a M2‐Like Alternative Activation State

The abundant presence of alternatively activated M2 macrophages within PDAC TME is widely recognized as a negative prognostic marker. Such immune cells contribute to tumor progression by inhibiting cytotoxic CD8^+^ T cells activity, secreting anti‐inflammatory cytokines, and supporting angiogenesis, thereby sustaining a protumoral and immunosuppressive environment.

To evaluate the impact of stromal components on macrophages polarization, 3D PDAC models were co‐cultured with macrophages, using a transwell insert system, which allowed paracrine signaling (Figure 3A). As a control, the obtained phenotypes were compared with those of CD14^+^ monocytes differentiated into prototypical M1, M2‐IL‐4, M2‐IL‐10, and M2‐IL‐6 macrophage subtypes. Our results demonstrate that macrophages co‐cultured with 3D tumor models exhibit low expression of M1 macrophage markers (CD86, CD80, CD274) and elevated expression of M2‐associated molecules (CD163, CD204). This expression profile is consistent across all 3D models but is more pronounced in STAMS and particularly in the cancer‐on‐a‐bead model (Figure 3C and Figure S3). These findings suggest that paracrine interactions between macrophages and 3D in vitro tumor models predominantly drive polarization toward an alternatively activated M2‐like macrophage phenotype.

In fact, the overexpression of immunomodulatory and stromal factors, such as TGF‐β1, CXC12, IL‐6, IL‐10, FAP, IDO, COX‐2, and PD‐L1 (Figure 2), previously demonstrated in 3D tumor‐stroma PDAC models provides a compelling mechanistic explanation for the observed polarization of macrophages toward an M2‐like macrophage phenotype. A growing body of evidence demonstrates the role of these factors in macrophage reprogramming within the native TME. TGF‐β and IL‐10 are classical anti‐inflammatory cytokines that suppress proinflammatory M1 functions and promote the acquisition of an immunosuppressive M2 phenotype [43, 44]. CXCL12, mainly secreted by CAFs, contributes to the recruitment of monocytes and macrophages to the tumor region as well as influences their polarization into a M2 tumor supportive state. In addition, the impairing of M1 polarization and promotion of M2 features is supported by IDO and COX‐2, both overexpressed in 3D PDAC models [45]. The combined action of these agents in tumor‐stroma 3D platforms creates a proper microenvironment that actively promotes the differentiation of macrophages into protumoral M2 macrophages, as evidenced by the upregulation of CD163 and CD204, and the downregulation of CD80, CD86, and CD274.

### 2.5 Impact of 3D Tumor‐Stroma Models in T cells Exhaustion

Aiming to further evaluate the immunological effects of the different 3D PDAC TMEs on T cell activation, viability and exhaustion, activated Jurkat T cells were directly co‐cultured with 3D PANC‐1 spheroids, STAMS or cancer‐on‐a‐bead models (Figure 3A). Analysis of T cell viability showed a significantly higher proportion of late apoptotic and necrotic Jurkat cells when co‐cultured with the different 3D in vitro platforms, compared to the conventional monolayer 2D cultures, suggesting that 3D TME and the associated secretome of PDAC 3D models induce a more evident immunosuppressive effect (Figure 3D and Figure S4). It was also verified that CD69 and CD279 (PD‐1) were significantly upregulated in Jurkat cells co‐cultured with 3D models, comparing to 2D cultures (Figure 3E). Such results indicate that interaction with 3D models cause exhaustion in Jurkat cells. Although CD69 is a well‐established early leukocyte activation marker [46], recent evidence suggests that its sustained or late expression is associated with T cell exhaustion, a phenomenon particularly evident in tumor‐infiltrating lymphocytes isolated from colorectal cancer, hepatocellular carcinoma, nonsmall cell lung cancer, and PDAC [47]. CD69 is also implicated in the regulation of Treg/Th17 differentiation, which contributes to exacerbation of immunosuppression in TME [48].

PD‐1 is a key inhibitory receptor driving T cell exhaustion in various tumor microenvironments, with its expression strongly correlated with impaired cytotoxic T cell function [49, 50]. The exhaustion profile is more pronounced in Jurkat co‐cultured with cancer‐on‐a‐bead models, which correlates with the observed highest percentage of late‐apoptotic cells, surpassing the levels observed in both PANC‐1 spheroids and STAMS. This suggests that despite compartmentalized cancer‐on‐a‐bead constructs may release immunomodulatory soluble factors at higher concentrations that result in a hyperstimulation of T cells, they ultimately exhibited a suppressed state, a cell marker in immunosuppressive TME. In fact, a higher upregulation of TGF‐β and PD‐L1, known to induce T cell dysfunction, was observed in cancer‐on‐a‐bead platforms, thus sustaining an immunosuppressive TME and providing a valuable system to study tumor‐induced T cell apoptosis and to screen potential immunotherapeutic strategies to sustain T cell function in hostile tumor microenvironments.

### 2.6 3D PDAC Models as Platforms for Immunotherapy Screening

**FIGURE 4:**
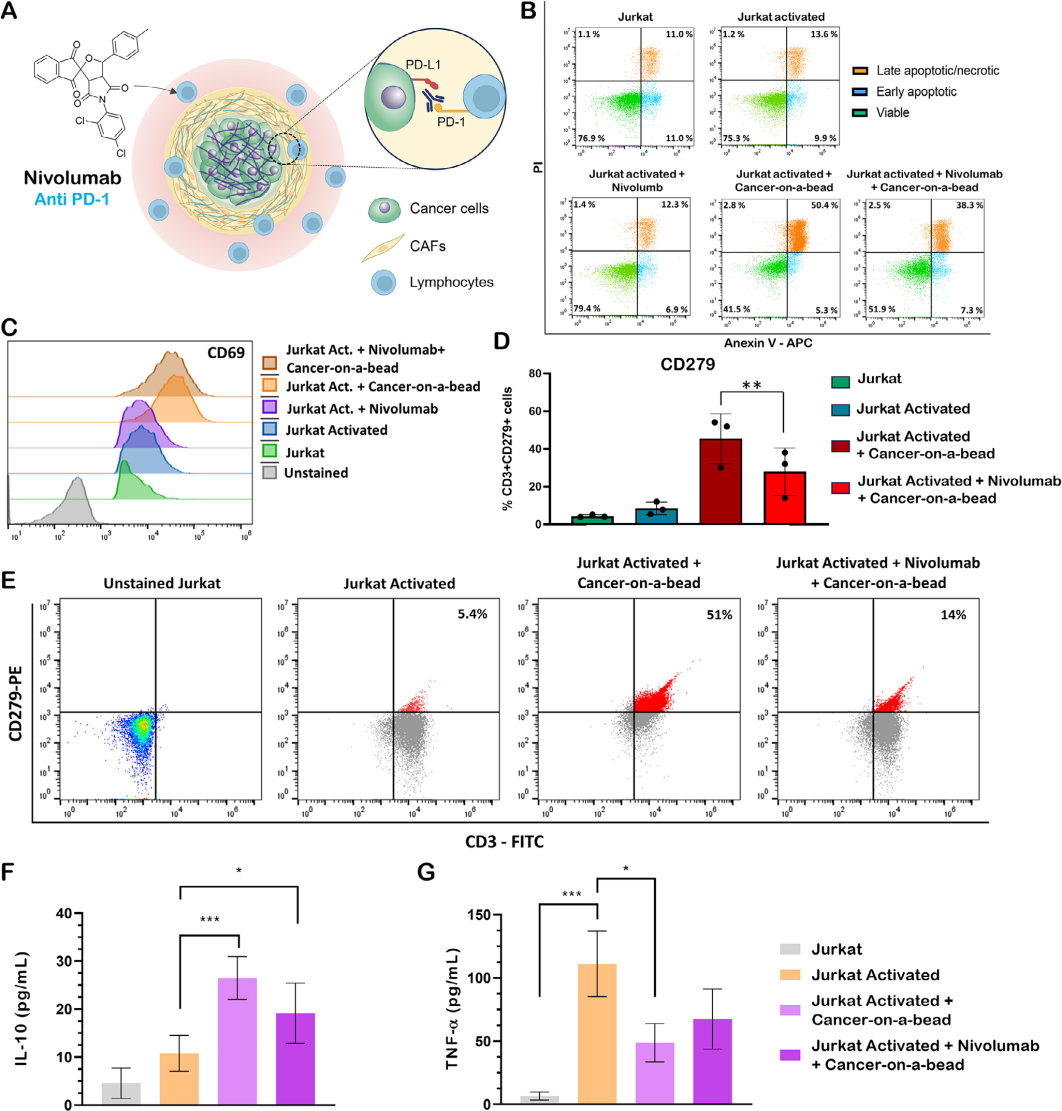
Evaluation of cancer‐on‐a‐beads relevance for screening anti PD‐1 immunotherapy. (A) Schematics of cancer‐on‐a‐bead models treatment with Nivolumab targeting cancer cells‐lymphocytes interaction. (B) Activated Jurkat cells were treated for 2 h with anti PD‐1 monoclonal antibody (Nivolumab) and then co‐cultured with cancer‐on‐a‐bead model for an additional 24 h in the continued presence of the antibody. T cells apoptosis/necrosis was analyzed with Annexin/PI staining by flow cytometry. The displayed dot plot represents one experiment, typical of three independent experiments conducted. (C–E) Using the same setup as described in (B), the expression of T cells activation marker CD69 (C) and exhaustion marker CD279 (D and E) markers were analyzed by flow cytometry. (F and G) ELISA‐based quantification of IL‐10 and TNF‐α in cell culture supernatant of the different conditions. The concentration of the different factors was normalized to total protein concentration. Data are presented as mean ± SD of at least three independent experiments. Statistical analysis was performed with student *t*‐test; **p < 0.01 Jurkat activated + Cancer‐on‐a‐bead versus Jurkat activated + Nivolumab + Cancer‐on‐a‐bead.

To evaluate the relevance of immunosuppressive cancer‐on‐a‐bead models to test immunotherapeutics, they were exposed to lymphocytes previously treated with Nivolumab, a monoclonal antibody targeting PD‐1 (Figure 4A). Following treatment and co‐culture, it was observed that there was a marked increase in the proportion of viable T cells and a reduction in late apoptotic/necrotic T cells compared to the nontreated counterpart, indicating a functional recovery of T cell survival and activation within the immunosuppressive 3D TME (Figure 4B). In addition, treated T cells co‐cultured with 3D platforms exhibited an elevated expression of CD69 and a reduced expression of CD279 (Figure 4C,D), indicating enhanced T cell activity, and suggesting the contribution of PD‐1/PD‐L1 signaling to T cell exhaustion in the untreated setting. To corroborate these results, IL‐10 and TNF‐α secretion in culture supernatants were analyzed by Enzyme‐Linked Immunosorbent Assay (ELISA). We observed that IL‐10 secretion increased when activated Jurkat cells are cultured with cancer‐on‐a‐bead models, consistent with the establishment of an immunosuppressive microenvironment, and decreased upon PD‐1 blockade (Figure 4F). Conversely, TNF‐α levels decreased when activated Jurkat cells are cultured with cancer‐on‐a‐bead models, reflecting suppression of proinflammatory T cells activity, and partially recovered following nivolumab administration, although not reaching the levels of activated Jurkat cells alone (Figure 4G). Overall, PD‐1 blockade derived from Nivolumab treatment effectively restored/increased T cells viability when directly co‐cultured with cancer‐on‐a‐bead models, mitigating the immunosuppressive effect exerted by the 3D in vitro microenvironment in T cells. These results showed that cancer‐on‐a‐bead models not only reproduced the characteristic immunosuppressive landscape of PDAC but also is a potential platform for testing new therapies targeting the immune system, such as immune checkpoint inhibitors. The improvements in T cell survival and activity following Nivolumab administration support the relevance of these models for evaluating T cell exhaustion and potential therapies to reverse this T cell state in pancreatic cancer.

## 3 Conclusions

PDAC remains one of the most lethal and treatment‐resistant malignancies, primarily due to its highly desmoplastic and immunosuppressive TME. This complex and rigid stroma not only impedes drug delivery and immune cell infiltration but also contributes to tumor progression through the dysregulation of mechano‐sensing pathways and promotion of an immune‐excluded phenotype. While immunotherapies represent a promising frontier in oncology, their clinical impact in PDAC has been minimal, underscoring the need for more predictive and physiologically relevant preclinical models. In this study, we demonstrate that bioengineered stratified 3D tumor‐stroma models can effectively recapitulate the structural, cellular, and immunosuppressive features of native PDAC. Through co‐culture with immune cell surrogates, these models reveal critical insights into tumor driven immune modulation and highlight the spatial and functional compartmentalization characteristic of the PDAC TME. These findings establish stratified 3D tumor models as robust platforms for interrogating tumor–immune interactions and evaluating immunotherapeutic strategies. By bridging the gap between in vitro systems and in vivo complexity, this approach holds significant promise for accelerating translational research and advancing precision medicine in PDAC.

## 4 Experimental Section

### 4.1 Materials

Ultralow adhesion (ULA) U‐bottom 96‐well plates (Corning, 7007), fetal bovine serum (FBS, E.U. approved, South America origin), Dulbecco's modified Eagle medium‐high glucose (DMEM‐HG), phosphate buffered saline, without Ca^2+^ and Mg^2+^ (D‐PBS, pH = 7.4), antibiotic‐antimycotic (ATB, Gibco – 10,000 U/mL of penicillin, 10,000 µg/mL of streptomycin, and 25 µg/mL of Amphotericin B), Calcein‐AM, propidium iodide (PI), and trinitrobenzene sulfonic acid (TNBS) were purchased from Thermofisher Scientific (Alfagene, Carcavelos, Portugal). Trypsin‐ethylenediaminetetraacetic acid (EDTA) detaching solution, gelatin form porcine skin type A, and hyaluronic acid (Molecular Weight (MW) ≈1.5 × 10^6^–1.8 × 10^6^ Da) were obtained from Laborspirit (Merck‐Sigma, Portugal). WX 2100 was purchased from Cytonix (Cytonix LLC, MD). MSC‐GRO Low serum, complete media was obtained from Vitrobiopharma, US. Nivolumab (anti‐PD‐1) was purchased from MedChemExpress, US.

### 4.2 Gelatin Methacrylate Synthesis

GelMA was produced in the previously described methodology. Gelatin was dissolved in a PBS solution at 50°C, to produce a 10% (w/v) solution. Thereafter, 0.6 mL of methacrylate anhydride/gram of gelatin was dropwise added under mild magnetic stirring. The reaction proceeded for 5 h, at 50°C, with constant magnetic stirring and protected from light. After that, the solution is centrifuged (3 min, 3500 g) to separate the nonreacted methacrylic anhydride. The supernatant comprising GelMA is then diluted with PBS and dialyzed (MW cut‐off 6–8 kDa) for 6 days. The purified GelMA is recovered by freeze drying (‐86°C, Telstar LyoQuest) for 7 days, protected from the light. The obtained degree of substitution was 85.2 ± 1.2%, as determined by the TNBS assay.

### 4.3 Hyaluronic Acid Methacrylate Synthesis

HAMA was synthesized by reacting high molecular weight hyaluronic acid (HA, MW ≈ 1.5 × 10^6^–1.8 × 10^6 ^Da) with glycidyl methacrylate as previously described. In brief, HA (2.0 g) was dissolved in filtered ultrapure water (200 mL, Milli‐Q water), under magnetic stirring, at room temperature (RT), to produce a 1% w/v working solution. Afterward, glycidyl methacrylate (35.5 mL) and triethylamine (25.3 mL) were dropwise added to *N*,*N*‐ dimethylformamide (DMF), in a schott flask under magnetic stirring. The organic DMF phase was added to HA aqueous phase, and the reaction occurred for 72 h, protected from the light. HAMA solution was then carefully transferred to a dialysis tubing (MWCO: 6–8 kDa) and dialyzed for 5 days, at RT, by using double distilled deionized water as dialysant. The purified polymer was then freeze‐dried (−86°C), for 7 days, in the dark. The modification degree was determined through ^1^H NMR spectroscopy as described in the literature. The obtained degree of substitution was D.S.: 19.4% ± 1.8%.

### 4.4 Fabrication of Superhydrophobic Surfaces

The production of superhydrophobic surfaces was performed as previously described. In brief, circular polystyrene 90 mm petri dish plates were spray coated with a FluoroThane‐MW reagent and left to dry overnight in a chemical safety fume hood. In the following day, the petri dish surface was washed several times with ethanol 70% (v/v) and oven dried at 37°C, for 24 h.

### 4.5 Culture of Cell Lines

Human pancreatic cancer cells (PANC‐1, ATCC CRL‐1469) were cultured in DMEM‐HG supplemented with sodium bicarbonate (3.7 g/L), 10% heat‐inactivated Fetal bovine serum (FBS), and 1% antibiotic/antimycotic. Human Pancreatic CAF‐Stellate Cells (CAF08, VitroBiopharma, USA) were cultured in MSC‐GRO Low serum, Complete Media, supplemented with 1% antibiotic/antimycotic, as recommended by the manufacturer. Both cancer and stromal cells were cultured in cell culture treated T‐flasks, and maintained under 5% CO_2_ atmosphere, at 37°C. The medium was replaced every 2–3 days. Jurkat (ATCC No TIB‐152, Manassas, VA) a human T‐cell line was cultured in RPMI 1640 (Gibco, ThermoScientific, Waltham, MA) supplemented with 1 mm pyruvate, 2 mm of glutamine, 100 U/mL penicillin, 100 µg/mL streptomycin, and 10% heat‐inactivated FBS (Gibco) (complete RPMI 1640). Cells were maintained in a confluence between 0.2 and 1 × 10^6^ cells/mL and mycoplasma contamination was routinely monitored.

### 4.6 Isolation of Human Monocytes and Differentiation of Dendritic Cells and Macrophages

Dendritic cells (DCs) and macrophages were differentiated from CD14^+^ monocytes. For this peripheral blood mononuclear cells (PBMCs) were isolated from buffy coats of healthy donors using Ficoll–Paque gradient centrifugation (GE Healthcare, Chalfont St. Giles, UK). The buffy coats were obtained from the Portuguese Blood and Transplantation Institute (IPST) under a protocol that grants access for academic research purposes only. These samples were not collected specifically for this study and were provided anonymously, with no personal donor information disclosed. Monocytes were isolated by positive selection using CD14 antibody‐conjugated magnetic beads (Miltenyi Biotec), according to the manufacturer's instructions. For DCs differentiation, monocytes were cultured at a density of 1 × 10^6^ cells/mL in complete RPMI 1640 supplemented with 250 U/mL of IL‐4 (Peprotech, London, UK) and 400 U/mL of granulocyte‐macrophage colony‐stimulating factor (GM‐CSF) (Peprotech). Half of the medium was renewed every 2 days and immature DCs (iDCs) were obtained on day 6 of culture. DCs maturation was induced by adding 50 ng/mL of tumor necrosis factor (TNF)‐α (Biolegend) and 25 ng/mL of IL‐1β (Biolegend) during 24 h. For differentiation of M1 macrophages, CD14^+^ monocytes were incubated at a density of 1 × 10^6^ cells/mL in complete RPMI 1640 supplemented with 50 ng/mL GM‐CSF for 7 days, followed by 48 h stimulation with 50 ng/mL of Interferon (IFN)‐γ (Peprotech) and 20 ng/mL lipopolysaccharide (LPS) (Sigma‐Aldrich). The alternative activated M2 macrophages were differentiated by culturing 1 × 10^6^ CD14^+^ monocytes in 1 mL of complete RPMI 1640 supplemented with 50 ng/mL M‐CSF for 7 days. Polarization in the different M2 subtypes was then achieved by incubation for additional 48 h with 20 ng/mL IL‐4 (M2‐IL‐4), 20 ng/mL IL‐10 (M2‐IL‐10), or 20 ng/mL IL‐6 (M2‐IL‐6).

### 4.7 3D PDAC Models Assembly

The generation of monotypic spheroids (i.e., comprising cancer cells only) and heterotypic stratified spheroids, STAMs, (i.e., comprising cancer cells and CAFs in a compartmentalized core–shell architecture) was performed as previously described [17,51]. Both 3D models were generated by the liquid overlay technique (LOT) using 96‐well ULA plates that promote cellular self‐aggregation into a scaffold‐free microtumor. PANC‐1 and CAFs were detached with trypsin and resuspended in culture medium. For assembling monotypic PANC‐1 spheroids, a cellular suspension (i.e., 5 × 10^4^ cells per well) was prepared and seeded into 96‐well ULA plates to generate 3D spheroids via LOT. The heterotypic 3D STAMS were produced through a sequential two‐stage procedure. In the first stage of assembly, a 3D spheroid core comprising 1 × 10^4^ PANC‐1 cells was maturated for 6 days in in vitro culture. At day 6 of tumor core culture, a suspension of human pancreatic stellate CAFs (i.e., 4 × 10^4^ cells per well) was seeded in the wells containing PANC‐1 3D spheroids to establish the stratified model. The generation of compartmentalized cancer‐on‐a‐bead models was carried out as previously outlined [18, 51]. In brief, their performance involved the use of a mechanical electronic repeater pipette (Eppendorf M4, Eppendorf, VWR) and superhydrophobic surfaces. PANC‐1 and CAFs were detached with trypsin and resuspended in culture medium. For assembly tumor core beads, a PANC‐1 cellular suspension (i.e., 1 × 10^4^ cells per µL) was prepared and centrifugate. The resulting pellet was resuspended in the GelMA‐based hydrogel, prepared by dissolving GelMA at 5% (w/v) in a sterile DPBS solution containing lithium phenyl‐2,4,6‐trimethylbenzoylphosphinate (LAP) photoinitiator at 0.1% (v/v). Tumor core beads (1 µL) were dispensed over the superhydrophobic surface and photocrosslinked (15 s, LED 406 nm, 9.25 mW cm^−2^). Then, stroma shell compartments (2 µL) combining CAFs laden in a GelMA 5% (w/v)‐HAMA 1% (w/v) ‐based hydrogel at a density of 2 × 10^4^ cells per µL, were dispensed above the core bead to generate the compartmentalized system. The stratified tumor‐stroma platform was then photocrosslinked (60 s, LED 406 nm, 9.25 mW cm^−2^) and the obtained living units were transferred to 96‐well round bottom ULA plates to maturate for 14 days. All the generated 3D PDAC models were maintained under a 5% CO_2_ atmosphere at 37°C and the culture medium was replaced at each 3 days of culture.

### 4.8 Live/Dead Analysis in 3D Tumor Models

After 3, 7, and 14 d of culture, a live/dead cell assay was performed for viability assessment. Briefly, 3D pancreatic tumor models were incubated with Calcein‐AM (5 µg/mL) and PI (2 µg/mL) in PBS at standard culture conditions (5% CO_2_ at 37°C), for 30 min. After washing with PBS, the 3D tumor models were observed under a widefield fluorescence microscope (Axio Imager M2, Carl Zeiss, Germany).

### 4.9 qPCR analysis

Total RNA was isolated from 2D and 3D tumor models with NZYol reagent (Nzytech, Lisbon, Portugal) according to the manufacturer's instructions and stored in RNA Storage Solution (Ambion, Foster City, CA) at −80°C until use. The RNA concentration and the levels of protein, salt, and organic solvent contamination were assessed using a NanoDrop spectrophotometer (Thermo Scientific, Wilmington, DE). RNA quantity was determined by measuring absorbance at 260 nm (OD260), while OD260/OD280 and OD260/OD230 ratios were used to evaluate protein and salt/organic solvent contamination, respectively. One microgram of total RNA was reverse transcribed with NZY First‐Strand cDNA Synthesis Kit (Nzytech, Lisbon, Portugal) and qPCR reactions performed in duplicate for each sample on a Bio‐Rad CFX Connect (Hercules, CA) using SYBR Green chemistry (NZYSupreme qPCR Green Master Mix). Transcription levels for indicated genes were analyzed with GenEx software version 7 (MultiD Analyses AB, Göteberg, Sweden) using *HPRT1* as reference gene. Primer sequences (Table S1) were designed using Beacon Designer software version 8.21 (Premier Biosoft International, Palo Alto, CA) and thoroughly tested.

### 4.10 Co‐culture of DCs, Macrophages, and Lymphocytes with Tumor Models

To address the immunomodulatory abilities of the different 2D and 3D tumor models, co‐culture assays were performed with DCs, macrophages, and T lymphocytes. iDCs were seeded in 12‐well plates at 1 × 10^6^ cells/well, allowed to stabilize for 2 h and then co‐cultured with 20 3D tumor structures or equivalent number of PANC‐1 cells through transwell membrane inserts (cellQART 12‐Well Insert 1.0 µm PET, SABEU GmbH). iDCs and 3D models/2D tumor cells were co‐cultured during 24 h followed by addition of 50 ng/mL of TNF‐α and 25 ng/mL of IL‐1β to the bottom of the well to promote DC maturation. After 24 h, DCs were collected, and phenotype analyzed by flow cytometry. To address the impact of tumor models on macrophage polarization, M0 macrophages were co‐cultured through transwell membrane inserts with 3D models/2D tumor cells for 48 h and the phenotype analyzed and compared to prototypical M1, M2‐IL‐4, M2‐IL‐10, M2‐IL‐6‐polarized cells. As we aimed to investigate the impact of tumors on lymphocyte exhaustion and viability—processes largely dependent on direct cell–cell interactions, such as PD‐1/PD‐L1 engagement—we performed direct co‐culture assays. For this, Jurkat were activated by treatment with 25 ng/mL PMA + 1 mg/mL ionomycin during 6 h and then directly co‐cultured with 3D models/2D tumor cells during additional 24 h. Following this period, activation and exhaustion markers CD69 and CD279 were analyzed by flow cytometry. Annexin/PI staining was also performed to address apoptotic/necrotic status of the lymphocytes. In selected experiments, following activation, Jurkat cells were treated with 10 µg/mL anti‐PD‐1 antibody (Nivolumab), for 2 h, then co‐cultured with the cancer‐on‐a‐bead tumor model for an additional 24 h in the continued presence of the antibody.

### 4.11 Flow Cytometry

At the end of co‐culture assays, DCs staining was performed using fluorescence‐conjugated antibodies for costimulatory/inhibitory molecules and maturation markers: CD40‐APC, CD86‐FITC, HLA‐ABC‐PE, HLA‐DR‐FITC (ImmunoTools GmbH), CD274‐PE (Pharmigen), and CD83‐APC (BD Biosciences). For phenotypic characterization of macrophages subtypes they were stained with CD80‐PE, CD86‐FITC, HLA‐DR‐APC (ImmunoTools GmbH), CD204‐PE (Biolegend), CD163‐AF647, CD206‐FITC (Pharmigen), and TREM2‐APC and Mertk‐FITC (Santa Cruz Biotechnology). Jurkat T lymphocytes were investigated for activation status with CD69‐APC and for exhaustion with CD279‐PE (both from ImmunoTools). Isotype‐matched antibodies were used as controls. Briefly, 0.2 × 10^6^ DCs, macrophages or Jurkat cells were washed and stained with the different fluorescence‐conjugated antibodies in PBS + 2% FBS (v/v) + 0,1% (v/v) sodium azide (FACS buffer) for 30 min, at 4°C, in the dark. Cells were subsequently washed twice in 1 mL cold PBS, resuspended in 300 µL FACS buffer and analyzed on an Accuri C6 flow cytometer (BD Bioscience, San Jose, CA). Jurkat cells were also assessed for apoptosis and necrosis using Annexin V‐APC (ImmunoTools) and propidium iodide (Merck KGaA), following the manufacturers’ instructions. Data were processed with FlowJo software (version 10) and results presented in dot plots as percentage of positive cells or in histograms as mean fluorescence intensity (MFI).

### 4.12 ELISA Assays

The quantification of soluble biomolecular markers secreted by the different PDAC models including IL‐10 (ab46059, Abcam, UK) and TNF‐α (ab181421, Abcam, UK) was performed by ELISA according to manufacturer's instructions. Absorbance was determined by using a multimodal Synergy HTX microplate reader (BioTek Instruments, USA).

### 4.13 Statistical Analysis

Statistical analysis was performed in GraphPad Prism 8 Software (Prism 8, trial version). One‐way analysis of variance (One‐way ANOVA) with Tukey's multiple comparisons was generally used for data analysis. A value of *p <* 0.05 was considered to be statistically significant.

## Conflicts of Interest

JFM and VM are co‐founders of Cellularis Biomodels company.

## Supporting information




**Supporting File**: adhm70530‐sup‐0001‐SuppMat.docx.

## Data Availability

The data that support the findings of this study are available from the corresponding author upon reasonable request.
