# Supplementary material for: Bioengineered Pancreatic Cancer Immunosuppressive Microenvironment Models for Screening Immunotherapies
Source: Adv Healthc Mater. 2025 Nov 28;15(8):e02758. doi: 10.1002/adhm.202502758 (PMC12927528; doi:10.1002/adhm.202502758)
Supplement: Supplementary file 1 — Supporting File: adhm70530‐sup‐0001‐SuppMat.docx. [file ADHM-15-0-s001.docx]

Supporting Information

Bioengineered Pancreatic Cancer Immunosupressive Microenvironment Models for Screening Immunotherapies

Maria V. Monteiro^1,2#^, Margarida Henriques-Pereira^1,2#^, Bruno M. Neves^3^, Vítor M. Gaspar^1,2,*^, João F. Mano^1,2,*^

**Table S1:** Primer sequences for targeted cDNAs.

| **Gene** | **RefSeq ID** | **Primers (F: forward; R; reverse)** |
| --- | --- | --- |
| *ABCB1* | NM_000927 | F: TCAGTCAAGTTCAGAGTCT  R: TAGCAAGGCAGTCAGTTA |
| *ABCC1* | NM_004996 | F: CAATCAATGCTGTTATTACT  R: CTCACTCTCAGTCTCTAT |
| *ABCG2* | NM_004827 | F: CTACAACTGGCTTAGACT  R: TGATGAATGGAGAAGATGA |
| *CCL2* | NM_002982 | F: TTTAGATACAGAGACTTGG  R: AAAGACCCTCAAAACATC |
| *CCL5* | NM_002985 | F: TGGAGATGAGCTAGGATGGA  R: TAGGACAAGAGCAAGCAGAA |
| *CD24* | NM_013230 | F: ATGATGAATGAGAATCTACC  R: TAGTGCTCTTGTTGAATG |
| *CD44* | NM_000610 | F: ATATCAGAGGAGTAGGAGAG  R: GTAACAATAACAGTGGTCATT |
| *CD133* | NM_001145847 | F: CAACGAGTCCTTCCTATA  R: CTCTCCAACAATCCATTC |
| *CD274* | NM_014143 | F: TCTTATTATGCCTTGGTGTAG  R: TGTGTATCACTTTGCTTCTT |
| *CXCL8* | NM_000584 | F: CACAGAAATTATTGTAAAGC  R: GATACCACAGAGAATGAA |
| *CXCL12* | NM_199168 | F: GCCTCTGAAGCCTATGTA  R: TAGTGGCAAGATGATGGT |
| *FAP* | NM_004460 | F:CTATGACCTTAGCAATGGAGAATT  R: AACAGGCGACCAGCATAA |
| *HPRT1* | NM_000194 | F: GCCATCTGCTTAGTAGAG  R: CTCAATAGTGCTGTGGTT |
| *IDO1* | NM_002164 | F: CCTGACTTATGAGAACAT  R: ATTGCCTTGAATACAGTA |
| *IL6* | NM_000600 | F: GGATTCAATGAGGAGACTT  R: ATCTGTTCTGGAGGTACT |
| *IL10* | NM_000572 | F: GTGGAGCAGGTGAAGAAT  R: TCTATGTAGTTGATGAAGATGTC |
| *MMP9* | NM_004994 | F: GGCAGATTCCAAACCTTT  R: GCAAGTCTTCCGAGTAGT |
| *NANOG* | NM_024865 | F: TGATGAAGATGTATTCGTATTG  R: AGATTAGCACAACCAACA |
| *POU5F1* | NM_002701 | F: GGTGAAGTTCAATGATGCT  R: TGTGTCTATCTACTGTGTCC |
| *PTGS2* | NM_000963 | F: TCAGCCATACAGCAAATC  R: CGCACTTATACTGGTCAA |
| *SOX2* | NM_003106 | F: TTCAAGGAGAGGCTTCTT  R: AACAAGACCACAGAGATG |
| *TGFB1* | NM_000660 | F: GGAAACCCACAACGAAATC  R: GCTCTGATGTGTTGAAGAAC |
| *VEGFA* | NM_001025366 | F: AATGTGAATGCAGACCAA  R: ACGCTCCAGGACTTATAC |


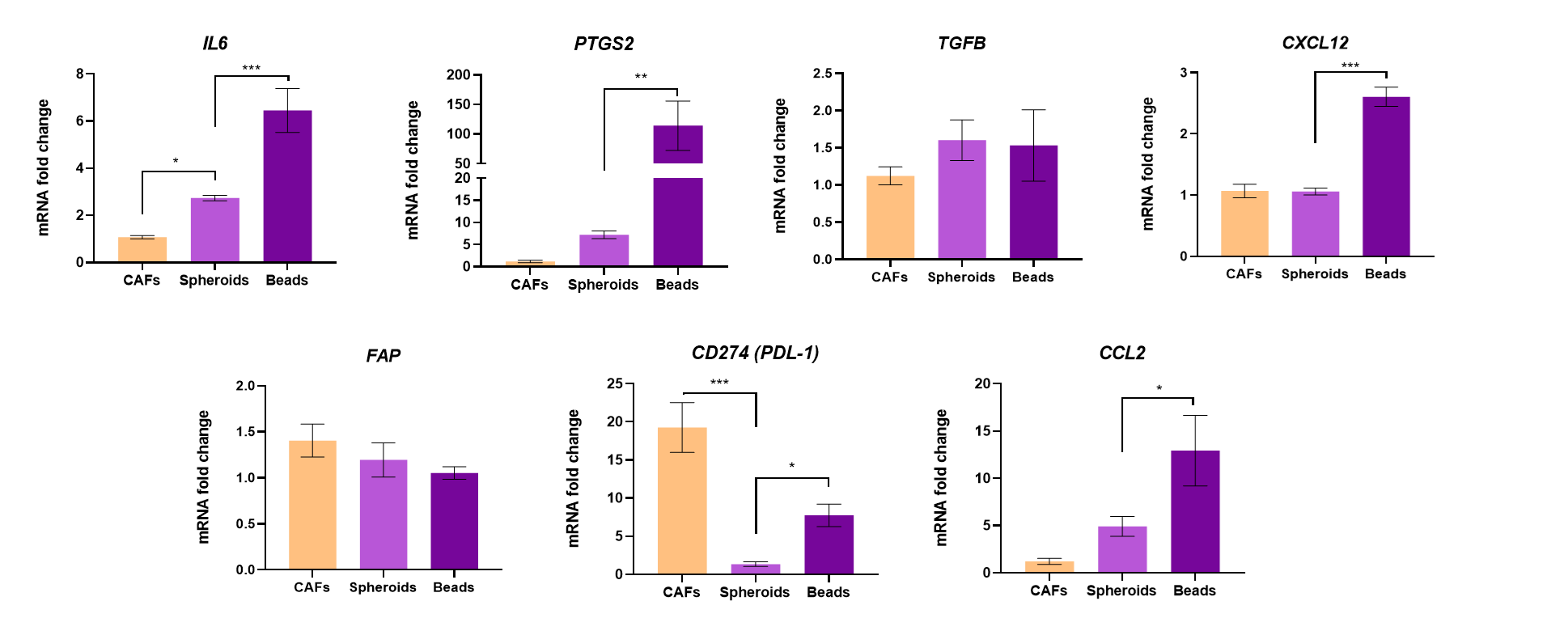


**Figure S1.** Differential expression of immunomodulatory and stromal genes in CAFs cultured under distinct configurations. Quantitative RT-PCR analysis of IL-6, COX-2 (PTGS2), CXCL12, TGF-β, CCL2, FAP, and PD-L1 in CAFs cultured as 2D monolayers, spheroids, or embedded within GelMA–HAMA hydrogel beads. Total mRNA was extracted from the different models, reverse transcribed, and the transcriptional levels of key immunoregulatory and stemness-related genes were analyzed by quantitative PCR (qPCR). Log2 mRNA fold changes for each gene were calculated relative to the maximum transcription levels observed across analyzed samples after normalization with reference gene HPRT1. The heatmap displays the mean values from three independent experiments.


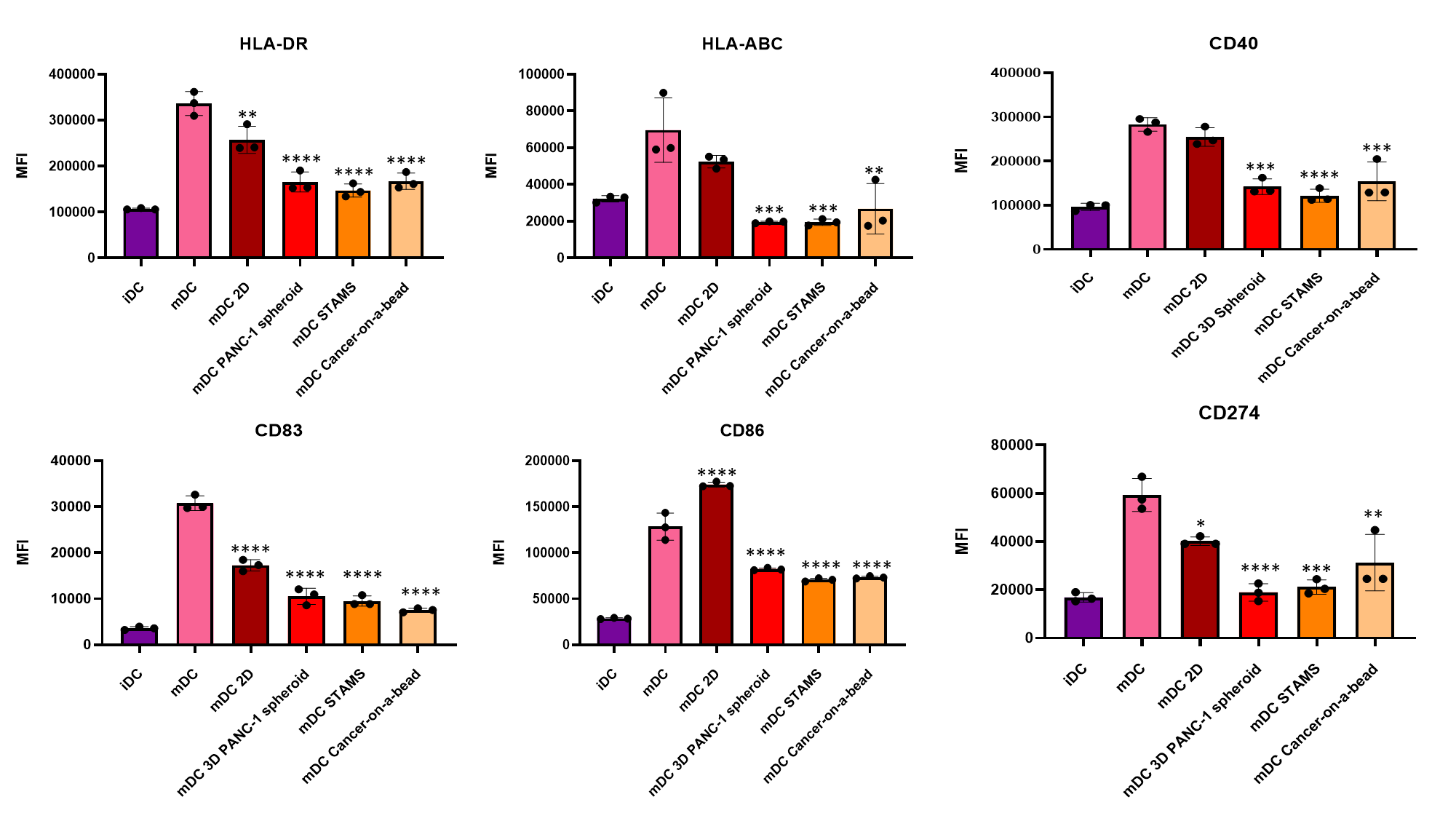


**Figure S2.** Evaluation of immunomodulatory effects of tumor-stroma 3D *in vitro* models over DCs. Immature DCs were cocultured with different tumor models for 24h and then IL-1β and TNF-α were added to the system for additional 24h to induce DC maturation. DC expression of maturation markers HLA-DR, HLA-ABC, co-stimulatory CD40, CD83, CD86 and co inhibitory CD274 were analyzed by flow cytometry. Results are presented as the mean ± SD of at least 3 independent experiments. Comparison of mDCs vs different tumor models was made by one-way analysis of variance (ANOVA) followed by Dunnett’s multiple comparison test; ^*^*P* <0.05, ^**^*P* <0.01, ^***^*P* <0.001 and ^****^*P* <0.0001


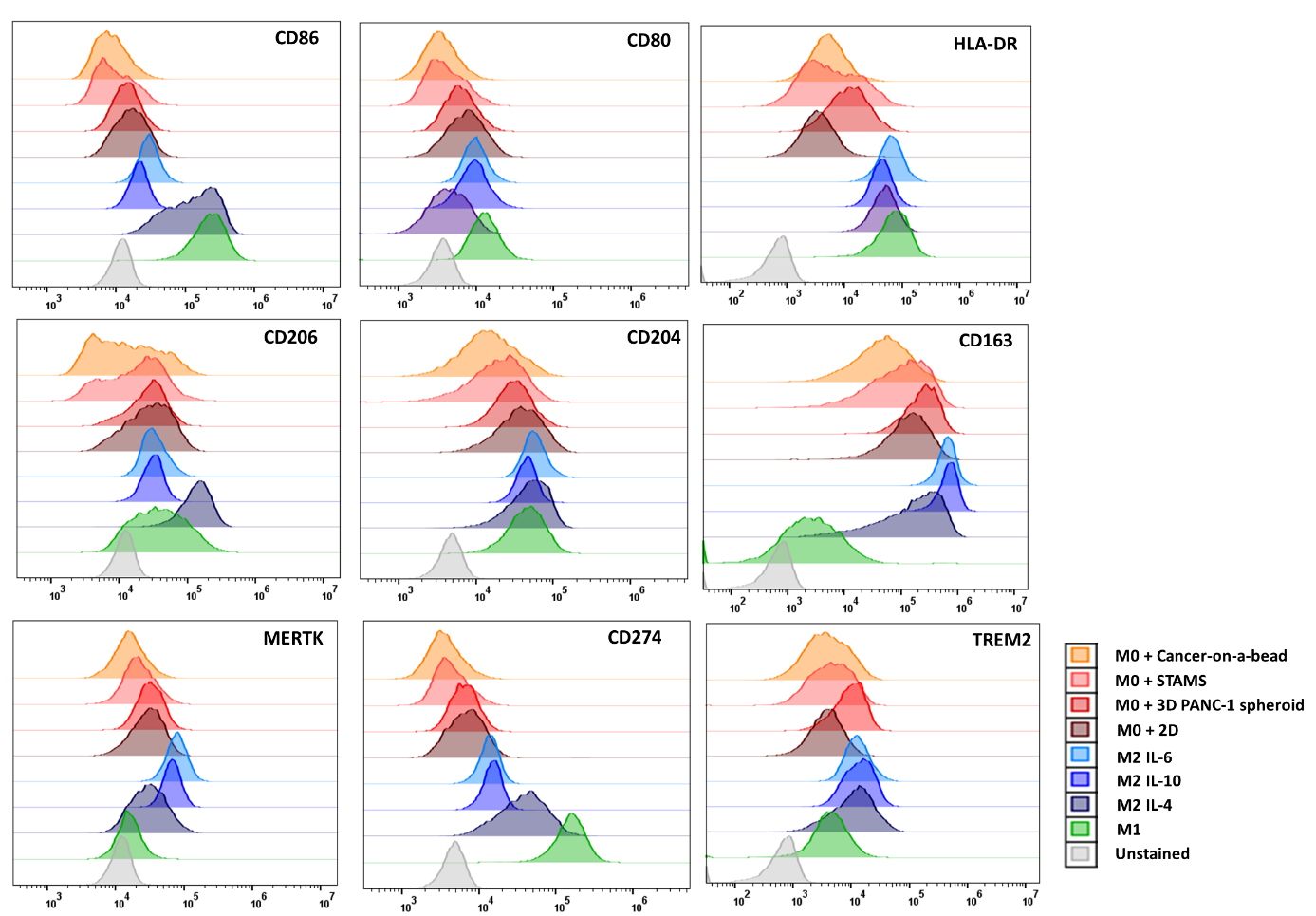


Figure S3. Phenotypic characterization of macrophages co-cultured with 3D PDAC models. Analysis of macrophages polarization phenotype. M0 macrophages were cocultured with the different tumor models for 48h and then the expression of key M1 and M2 markers were analyzed by flow cytometry. The phenotype is compared to prototypical M1, M2-IL-4, M2-IL-10, M2-IL-6 -polarized cells. The displayed histograms represent one experiment, typical of three independent experiments conducted.


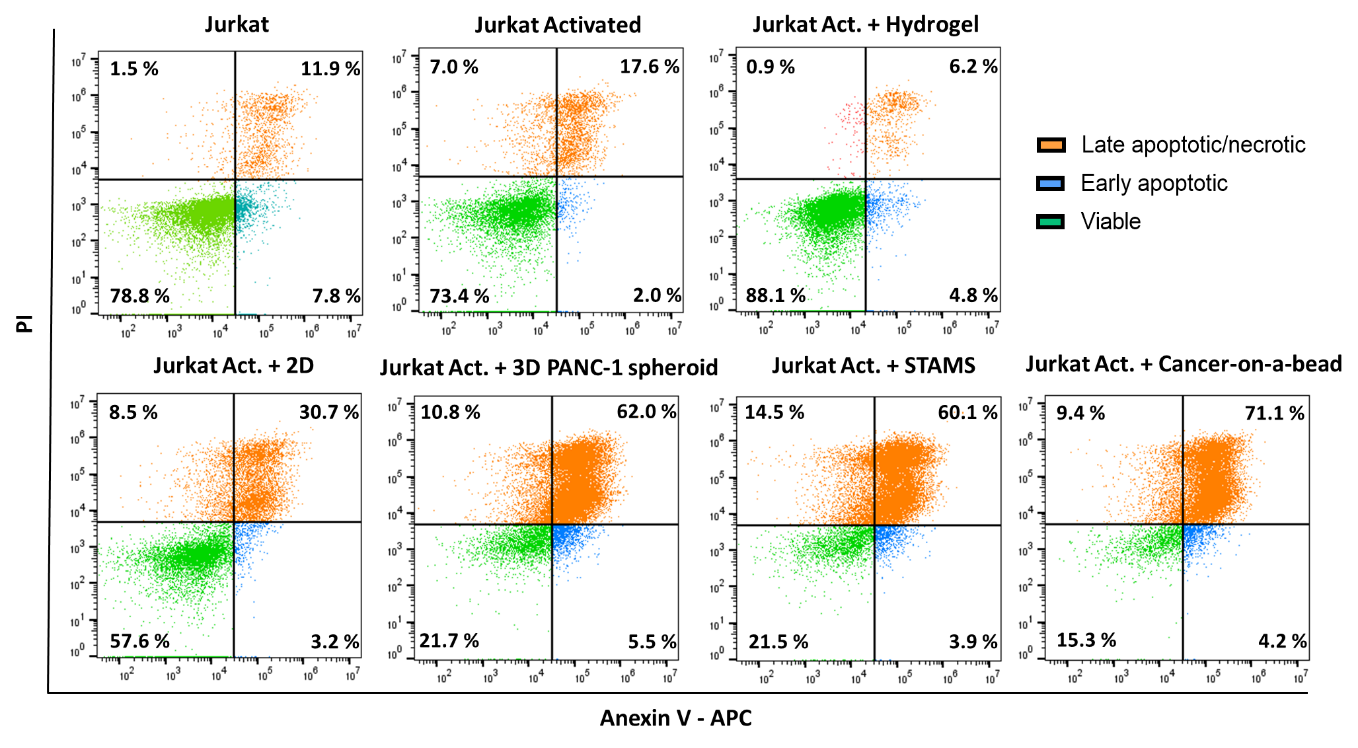


**Figure S4.** Impact of tumours models on lymphocyte viability. Activated Jurkat cells were co-cultured with different PDAC platforms and apoptotic/necrotic state was analyzed with Annexin / PI staining by flow. The displayed dot plots represent one experiment, typical of three independent experiments conducted.
